# Exploring YAP1-related TIME in SCLC: implications for survival and treatment response to immuno-chemotherapy

**DOI:** 10.20517/cdr.2024.177

**Published:** 2025-02-17

**Authors:** Yu-Qing Chen, Jia-Xiong Tan, Ling-Ling Gao, Jia-Xing Yang, Jie Huang, Jin-Ji Yang, Qiang Zhao

**Affiliations:** ^1^Tianjin Medical University Cancer Institute and Hospital, National Clinical Research Center for Cancer, Tianjin 300060, China.; ^2^Tianjin’s Clinical Research Center for Cancer, Tianjin 300060, China.; ^3^Key Laboratory of Cancer Prevention and Therapy, Tianjin 300060, China.; ^4^Beihang University, Beijing 100191, China.; ^5^Guangdong Lung Cancer Institute, Guangdong Provincial Key Laboratory of Translational Medicine in Lung Cancer, Guangdong Provincial People’s Hospital (Guangdong Academy of Medical Sciences), Southern Medical University, Guangzhou 510080, Guangdong, China.; ^6^Department of Pediatric Oncology, Tianjin Medical University Cancer Institute and Hospital, National Clinical Research Center for Cancer, Tianjin 300060, China.; ^#^Authors contributed equally.

**Keywords:** Small-cell lung cancer, YAP1, immunotherapy, tumor immune microenvironment, CD4 T cell

## Abstract

**Aim:** Small-cell lung cancer (SCLC) is usually diagnosed as an advanced stage with a poor outcome. SCLC has limited response to immunotherapy due to the absence or lack of immune cell infiltration, so studying its tumor immune microenvironment (TIME) is essential.

**Methods:** The study involved patients with extensive-stage small-cell lung cancer (ES-SCLC) diagnosed at the Guangdong Lung Cancer Institute between January 2018 and April 2022 who had received the atezolizumab/carboplatin/etoposide (ECT) treatment. We used multi-immunohistochemistry (mIHC) to assess the prognostic value of YAP1 and TIME in SCLC, with results confirmed using public data.

**Results:** 15 patients with sufficient baseline biopsy samples were included in this study. The total population of YAP1-positive cells is inversely related to progression-free survival (PFS) and shows a potential negative correlation with overall survival (OS). CD56-positive cells are the primary components of TIME in SCLC tumor parenchyma and stroma. The total population and cell density of YAP1-positive cells are significantly positively correlated with CD4-positive cells. Furthermore, in the tumor parenchyma, both the proportion and the cell density of YAP1-positive cells are positively correlated with that of FOXP3-positive cells. The total population of CD56-positive cells showed a negative correlation trend with YAP1-positive cells but without significant difference.

**Conclusion:** YAP1 has shown prognostic value in SCLC patients receiving ECT regimen treatment. The high expression level of YAP1 seems related to the inhibitory TIME. However, some prospective studies with larger populations are warranted.

## INTRODUCTION

Worldwide, lung cancer is a highly aggressive and widespread illness, with an annual rise of more than 2.2 million cases and 1.8 million fatalities^[1]^. The combination of chemotherapy with targeted therapies for high-frequency mutations - such as epidermal growth factor receptor (EGFR), anaplastic lymphoma kinase (ALK), and ROS proto-oncogene 1 (ROS1) - along with immunotherapeutic agents targeting the programmed cell death 1/programmed cell death-ligand 1 (PD-1/PD-L1) axis, has culminated in an objective response rate exceeding 83% in patients with non-small-cell lung cancer (NSCLC)^[2,3]^. However, small-cell lung cancer (SCLC), another pathological tissue type, is mostly diagnosed as extensive-stage small-cell lung cancer (ES-SCLC), with a poor prognosis, and the median 5-year overall survival (OS) was 7 months according to data from the US Surveillance, Epidemiology, and End Results (SEER) database^[4,5]^. SCLC is classified into pure SCLC and combined SCLC, the latter of which includes any proportion of NSCLC components or a large-cell neuroendocrine carcinoma (LCNEC) component exceeding 10%, as defined by the 2022 National Comprehensive Cancer Network (NCCN) SCLC guidelines^[6]^. Currently, the treatment of SCLC has also entered the era of immunotherapy, as the NCCN guidelines recommend first-line chemo-immunotherapy for ES-SCLC patients^[7]^. In the IMpower133 study, the application of Atezolizumab in combination with carboplatin and etoposide (ECT) as a first-line treatment for patients with ES-SCLC has led to a significant enhancement in both OS and progression-free survival (PFS), as compared to the use of a placebo in conjunction with ECT, that confirm the role of Atezolizumab in first-line immunotherapy for ES-SCLS^[8,9]^.

The tumor microenvironment (TME) is a coordinated cellular system that primarily includes immune cells, endothelial cells, fibroblasts that connect to the extracellular matrix, cytokines, chemokines, and various metabolites^[10,11]^. The TME, especially the tumor immune microenvironment (TIME), is associated with the survival outcomes of tumor patients. The recently developed classifier has demonstrated prognostic utility in The Cancer Genome Atlas (TCGA) dataset and predictive value in lung cancer. Specifically, patients with tumors predicted to exhibit CD8-inflamed phenotypes experienced extended OS compared to those with CD8-desert phenotypes. In the phase III OAK clinical trial, patients with NSCLC who received immune checkpoint inhibitor therapy exhibited a longer OS when their tumors were classified as CD8-inflamed, highlighting the potential of this classifier in stratifying patients for optimal therapeutic interventions^[12]^. Furthermore, the employment of multitargeted antiangiogenic tyrosine kinase inhibitors (TKIs) and nanomaterials in the therapeutic management of lung cancer has expanded the scope of targeting beyond traditional oncogenic drivers. This innovative approach allows for the simultaneous engagement of additional components of the TIME, encompassing cancer-associated fibroblasts (CAFs), tumor-associated macrophages (TAMs) and tumor-associated neutrophils (TANs)^[13-15]^.

Based on RNA gene transcriptomics, a series of characteristic genes have been identified to be associated with immuno-infiltration and are considered to be predictors of immunotherapy efficacy in SCLC^[16]^. Compared to lung adenocarcinoma, SCLC has less infiltration of immune cells and stronger immune isolation^[17]^. Although SCLC patients generally have high tumor mutation burden (TMB), the limited infiltration of immune cells in their TME, low expression of PD-L1, and lack of antigen presentation contribute to SCLC being defined as an immune “cold tumor”. This creates an inhibitory TIME, which restricts the effectiveness of immunotherapy^[18-20]^. Enhancing immune cell infiltration in SCLC represents a critical challenge that warrants further investigation and strategic intervention.

YAP1 is a principal effector of the Hippo signaling pathway that is involved in the regulation of tumor proliferation and migration^[21-23]^. Recent research has defined the dominant expression of transcription factor YAP1 as one of four subtypes of SCLC^[24]^. Furthermore, YAP1 has shown therapeutic vulnerability in SCLC^[25]^. Our previously analyzed results have indicated that YAP1 has potential predictive value for the efficacy of SCLC immunotherapy^[26]^. However, there is currently limited research on the impact of YAP-1 protein on the TIME and its possible related mechanisms. This study aims to explore the effects of YAP-1 on immune cell infiltration and the TME.

## METHODS

### Patients and clinical characteristics

The study involved patients with ES-SCLC diagnosed at the Guangdong Lung Cancer Institute from January 1, 2018, to April 30, 2022, who had received the ECT treatment. The baseline clinical and pathological data were analyzed retrospectively. The clinical staging is determined based on the Veterans Administration Lung Study Group staging system and the eighth edition of the American Joint Committee on Cancer tumor-node-metastasis staging system^[27,28]^. This research received approval from the Ethics Committee of Guangdong Provincial People’s Hospital.

### Response evaluation of treatment

Every two cycles of ECT treatment, patients underwent response evaluation using computed tomography (CT). According to the efficacy evaluation criteria of solid tumor (RECIST) version 1.1, assessments of complete remission (CR) or partial remission (PR) and disease stability (SD) or disease progression (PD) were conducted^[29]^. PFS was measured from the start of ECT treatment to either the date when the tumor progressed or the most recent follow-up. OS was calculated from the time of confirmed diagnosis to either the date of death or the latest follow-up. The last follow-up date was April 1, 2024.

### Multi-immunohistochemistry

Multi-immunohistochemistry (mIHC) was tested at Genecast Biotechnology Co., Ltd. (Beijing, China). Detect panel included YAP1, CD4, CD8, PanCK, FOXP3, and CD56. Conduct experiments using patient paraffin-embedded tissue specimens, with 2-3 slices μm thickness, followed by epitope extraction, non-specific protein antigen blocking, primary antibody incubation, secondary antibody incubation, and Tyramine signal amplification (TSA) staining and color development. CD8 primary antibodies (SP16, 1:100, Zsbio, China) were incubated overnight at 4 °C, while the remaining primary antibodies, including YAP-1 (ET1608-30, 1:100, Hua’an Biotech, China), CD4 (EP204, 1:100, Zsbio, China), PanCK (ZM0069, 1:100, Zsbio, China), FOXP3 (7H9-D6-A10, 1:100, Zsbio, China), and CD56 (UMAB83, 1:100, Zsbio, China), were incubated at room temperature for 1 h. Using Opal seven-color multi-color immunohistochemistry kit (NEL797B001KT, PerkinElmer, USA) for color development, including DAPI (4’, 6-diamidino-2-phenylindole) fluorescent dye, Opal 480, Opal 520, Opal 570, Opal 620, Opal 690, Opal 780, TSA fragrance system (NEL703001KT, PerkinElmer, USA), *etc.* Calculate the area of the specimen and the areas of the tumor and stromal regions and count the total number of cells in the specimen and each type of stained cell separately. Use the TissueFAX SL viewer multispectral image analysis software (TissueFAX SL viewer 7.1; TissueGnostics, China) for fluorescence signal recognition, image analysis, and image acquisition.

### Data sources

The FPKM values of transcriptome sequencing data and corresponding clinical information were obtained from a previously published article by George *et al.*, and the row expression data are available via the European Genome-phenome Archive (EGA) EGAS00001000925 (*n* = 81)^[30]^. The expression data were normalized and Log2 transformed.

### Immune infiltration landscape

The MeTIL scores were calculated using PCA, as previously reported^[31]^. Then, immune microenvironment infiltration was assessed using curated immune signatures, including 22 CIBERSORT signatures and two MCP counter signatures, with analysis performed through the GSVA R package^[32]^.

### Statistical analysis

The chi-square test or Fisher’s exact test was applied to compare inter-group differences. Kaplan-Meier curves were utilized to determine PFS and OS. The correlation coefficient is calculated using Spearman correlation. All statistical analyses were evaluated using GraphPad Prism 9.1.1 (GraphPad Software, Boston, USA), SPSS 22.0 software (SPSS, Inc., Chicago, IL, USA), and R (version 3.6.4). Two-sided *P* values < 0.05 were considered statistically significant.

## RESULTS

### Clinical and pathological characteristics

Patients with ES-SCLC who had received the ECT regimen were retrospectively enrolled, and we eventually collected biopsy specimens from 15 patients prior to their ECT treatment. The clinical baseline information is shown in Supplementary Table 1. P7 was diagnosed as SCLC combined with about 3% lung squamous cell carcinoma. All patients received ECT as first-line therapy except P2, who received a second-line ECT regimen after PD on EC chemotherapy. In our cohort, most patients were male (13/15, 86.7%) and smokers (12/15, 80%). There were no statistical differences in clinical characteristics, such as the serum NSE level and the Ki67 index, between the responder (CR/PR patients) group and the non-responder (SD/PD patients) group [Table 1].

**Table 1:** Clinical baseline characteristics

**Clinical features**	**Level**	**Responder (PR)** ***n* = 7**	**Non-responder (SD/PD)** ***n* = 8**	**Total** ***n* = 15**	***P*-value**
Age	Median (range)	66 (54-71)	63 (54-72)	64 (54-72)	0.224
Age group	≤ 65	2 (28.6%)	5 (62.5%)	7 (46.7%)	0.315
	> 65	5 (71.4%)	3 (37.5%)	8 (53.3%)	
Gender	Male	6 (85.7%)	7 (87.5%)	13 (86.7%)	1.000
	Female	1 (14.3%)	1 (12.5%)	2 (13.3%)	
Smoke	Smoker	5 (71.4%)	7 (87.5%)	12 (80.0%)	0.569
	Non-smoker	2 (28.6%)	1 (12.5%)	3 (20.0%)	
Type	Pure SCLC	7 (100.0%)	7 (87.5%)	14 (93.3%)	1.000
	Combined SCLC	0 (0.0%)	1 (12.5%)	1 (6.7%)	
Line of ECT	1st (ECT)	7 (100.0%)	7 (87.5%)	14 (93.3%)	1.000
	2st (ECT)	0 (0.0%)	1 (12.5%)	1 (6.7%)	
Ki67 index	< 90	4 (85.7%)	4 (50.0%)	8 (53.3%)	1.000
	≥ 90	3 (14.3%)	4 (50.0%)	7 (46.7%)	
NSE (ng/mL)	Median (range)	63.7 (16.13-155)	45.7 (21.4-370)	49.66 (16.13-370)	0.378

PR: Partial remission; SD: disease stability; PD: disease progression; SCLC: small-cell lung cancer; ECT: Atezolizumab in combination with carboplatin and etoposide; NSE: neuron-specific enolase.

### Immune landscape of different therapeutic effects

In our cohort, 7 patients achieved PR, 7 patients achieved SD, and one patient experienced PD after three cycles of ECT treatment. Among all, P5 discontinued the ECT treatment because of grade 3-4 increased level of γ-glutamyl transpeptidase (γ-GT) after 24 days of medication, and thus the best recorded treatment outcome was SD. The mPFS was 4.8 months (95%CI: 3.6-7.5) and the mOS was 12.0 months (95%CI: 9.0-20.5) [Supplementary Figure 1]. Two patients remained alive until the last follow-up.

**Figure 1:**
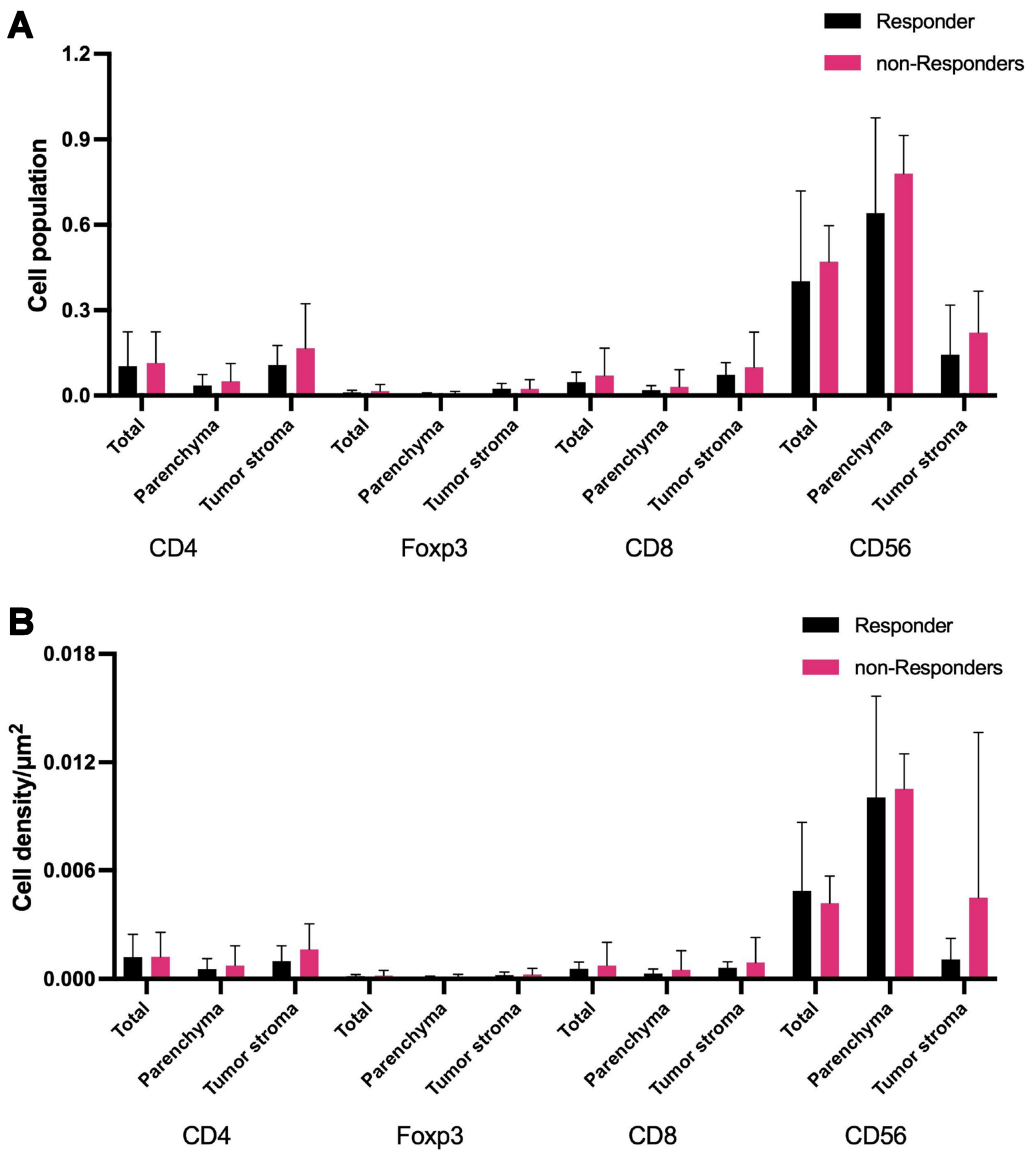
The cell proportion and cell density of various immune cells in ES-SCLC. Patients are grouped according to the efficacy of immunotherapy, and the tumor parenchyma and stromal regions are divided based on PanCK staining. (A) The proportion of various immune cells in tissue specimens; (B) The cell density of various immune cells in tissue specimens. ES-SCLC: Extensive-stage small-cell lung cancer.

We measured the area of the specimen and the total number of cells inside, and distinguished tumor cells and parenchymal cells based on PanCK staining. We calculated the area of the tumor parenchymal region and tumor interstitial region, as well as the number of tumor cells and parenchymal cells, respectively. Among all patients, the baseline biopsy specimen for ECT treatment in P3 was a pleural fluid sediment embedded specimen, which cannot be divided into tumor parenchymal and stromal regions. Therefore, this patient was included only for analysis of the overall proportion and density of each cell type. CD56-positive cells were the main components of the TIME in both tumor parenchyma and stroma. And except for the total cell density of CD56-positive cells (0.004/μm^2^ *vs.* 0.006/μm^2^, *P* = 0.308), the proportion and density of all types of immune cells in the non-responder group were higher than those in the responder group [Figure 1 and Supplementary Table 2]. Among them, in the tumor stroma, compared to those in the responder group, both the mean population and the cell density of CD4-positive cells were numerically higher in the non-responder group (0.166 *vs.* 0.108, *P* = 0.366 and 0.002/μm^2^ *vs.* 0.001/μm^2^, *P* = 0.351).

### Potential prognostic value of YAP1 in ES-SCLC

**Figure 2:**
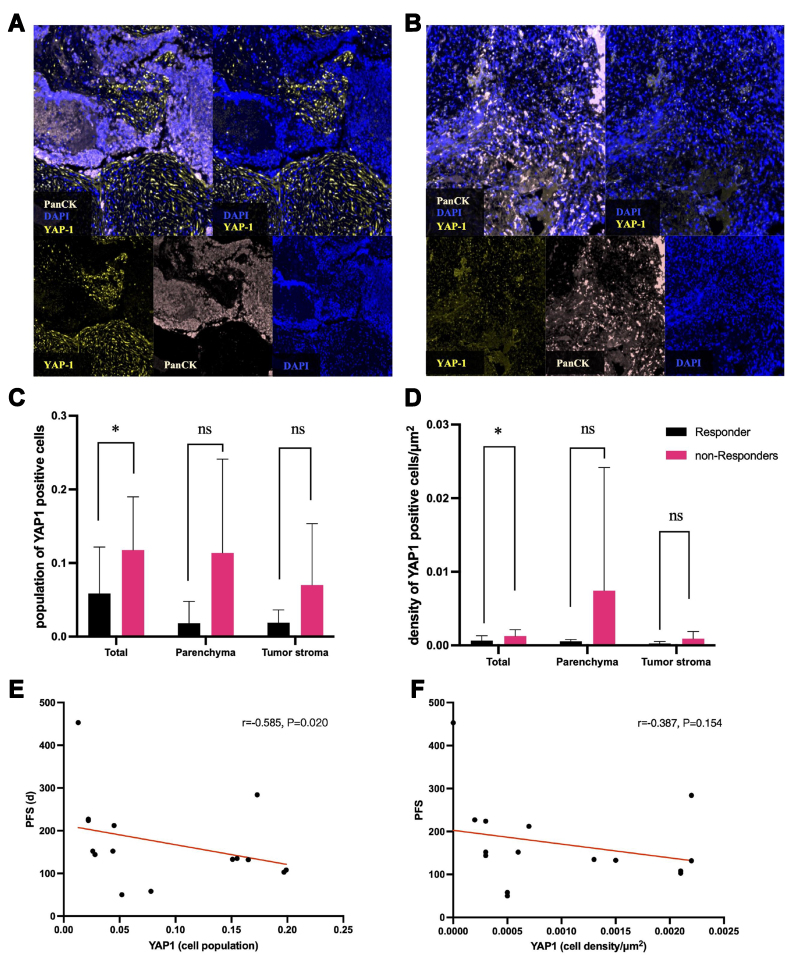
The distribution and prognostic value of YAP1-positive cells in ES-SCLC. (A) High-level YAP1-positive cells infiltrated; (B) Low-level YAP1-positive cells infiltrated. The fluorescence signal channels corresponding to each image are indicated in the bottom left corner of each figure. The tumor parenchyma and stromal regions are divided based on PanCK staining through mIHC; (C and D) The cell population and the cell density of YAP1-positive cells in responder and non-responder groups, as well as in tumor parenchyma and stroma, are shown. NS: *P* > 0.05, ^*^P < 0.05; (E and F) The linear regression fitting curve calculated by Spearman correlation between PFS and cell population and cell density of YAP1-positive cells, respectively. ES-SCLC: Extensive-stage small-cell lung cancer; mIHC: multi-immunohistochemistry; PFS: progression-free survival.

Overall, YAP1-positive cells were predominantly located in the tumor stroma rather than infiltrating the tumor parenchyma [Figure 2A-D]. The total population of YAP1-positive cells differed significantly between the responder and non-responder groups (mean cell populations: 0.035 *vs.* 0.118, *P* = 0.014). Additionally, the total cell density of YAP1-positive cells was significantly higher in the non-responder group compared to the responder group (mean cell densities: 0.0004/μm^2^ *vs.* 0.013/μm^2^, *P* = 0.028). However, no significant differences were observed in the YAP1-positive cell populations within the tumor stroma or parenchyma between the two groups (0.019 *vs.* 0.074, *P* = 0.075, and 0.018 *vs.* 0.130, *P* = 0.168, respectively). Similarly, no significant differences were found in YAP1-positive cell density in the tumor stroma or parenchyma (0/μm^2^ *vs.* 0.001/μm^2^, *P* = 0.112, and 0.001/μm^2^ *vs.* 0.008/μm^2^, *P* = 0.338, respectively). Nevertheless, both the population and density of YAP1-positive cells in the tumor stroma and parenchyma showed an increasing trend in the non-responder group. [Figure 2E and F, Supplementary Table 2].

As for the correlation of YAP1 expression and PFS, the total population of YAP1-positive cells was negatively correlated with PFS (*r* = -0.585, *P* = 0.023), while the cell density of YAP1 had no significant correlation with PFS (*r* = -0.387, *P* = 0.154). Moreover, both the total population and cell density of YAP1-positive cells showed trends of negative correlation with OS, though the associations were not statistically significant (*r* = -0.340, *P* = 0.216; *r* = -0.400, *P* = 0.140). Notably, the cell density of YAP1-positive cells in the tumor stroma was significantly correlated with OS (*r* = -0.552, *P* = 0.040). In contrast, the cell population of YAP1-positive cells in parenchyma was positively correlated with OS (*r* = 0.648, *P* = 0.017) [Supplementary Figure 2].

### The correlation between YAP1-positive cells and immune cell subsets

**Figure 3:**
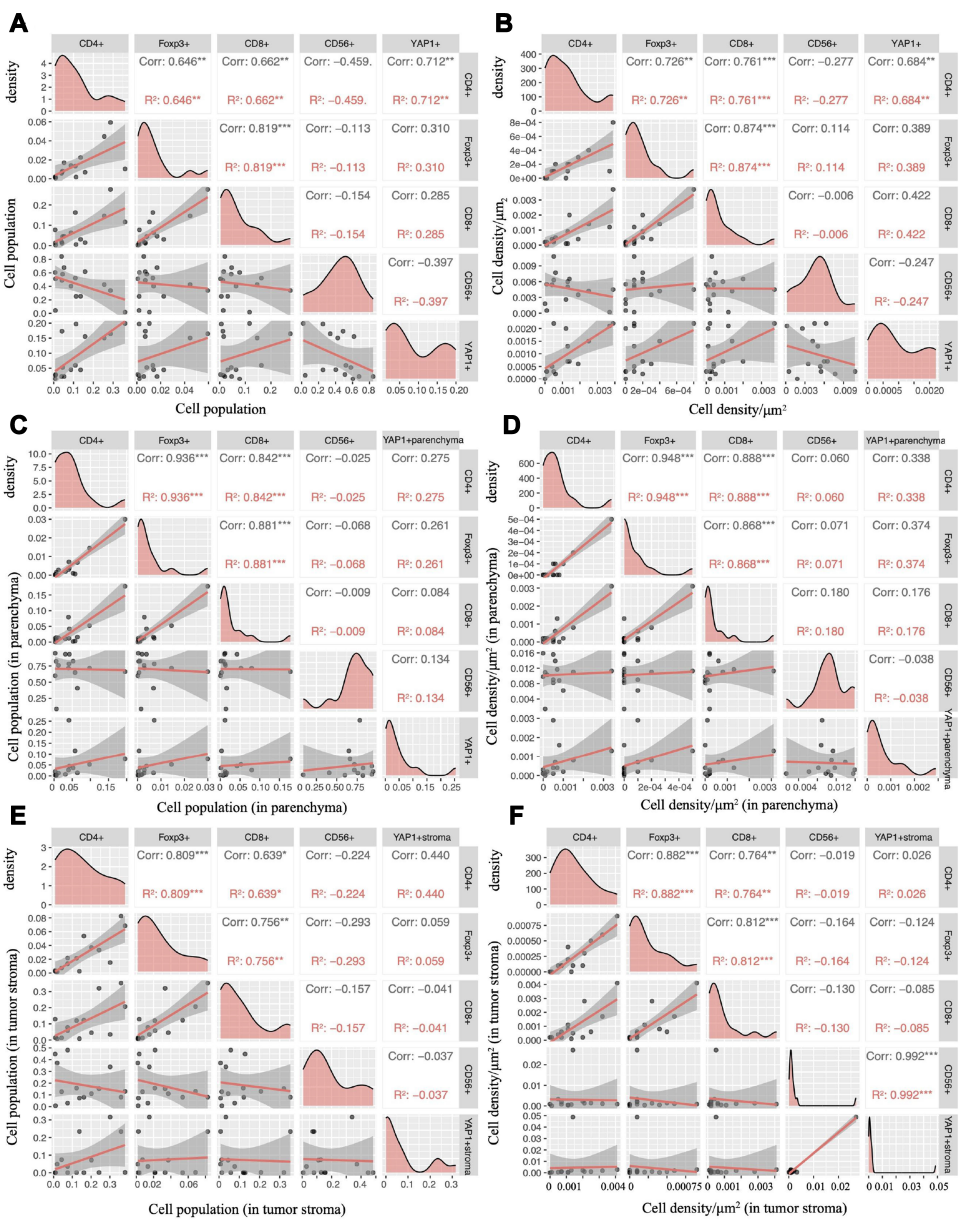
Correlation between infiltration of various immune cells and YAP1-positive cells in ES-SCLC. The correlation between the infiltration of CD4-, FOXP3-, CD8-, and CD56-positive immune cells and YAP1-positive cells was calculated using Spearman correlation for the following parameters: (A) Total cell population; (B) Total cell density; (C) Cell population in parenchyma; (D) Cell density in parenchyma; (E) Cell population in the tumor stroma; (F) Cell density in the tumor stroma. ES-SCLC: Extensive-stage small-cell lung cancer.

**Figure 4:**
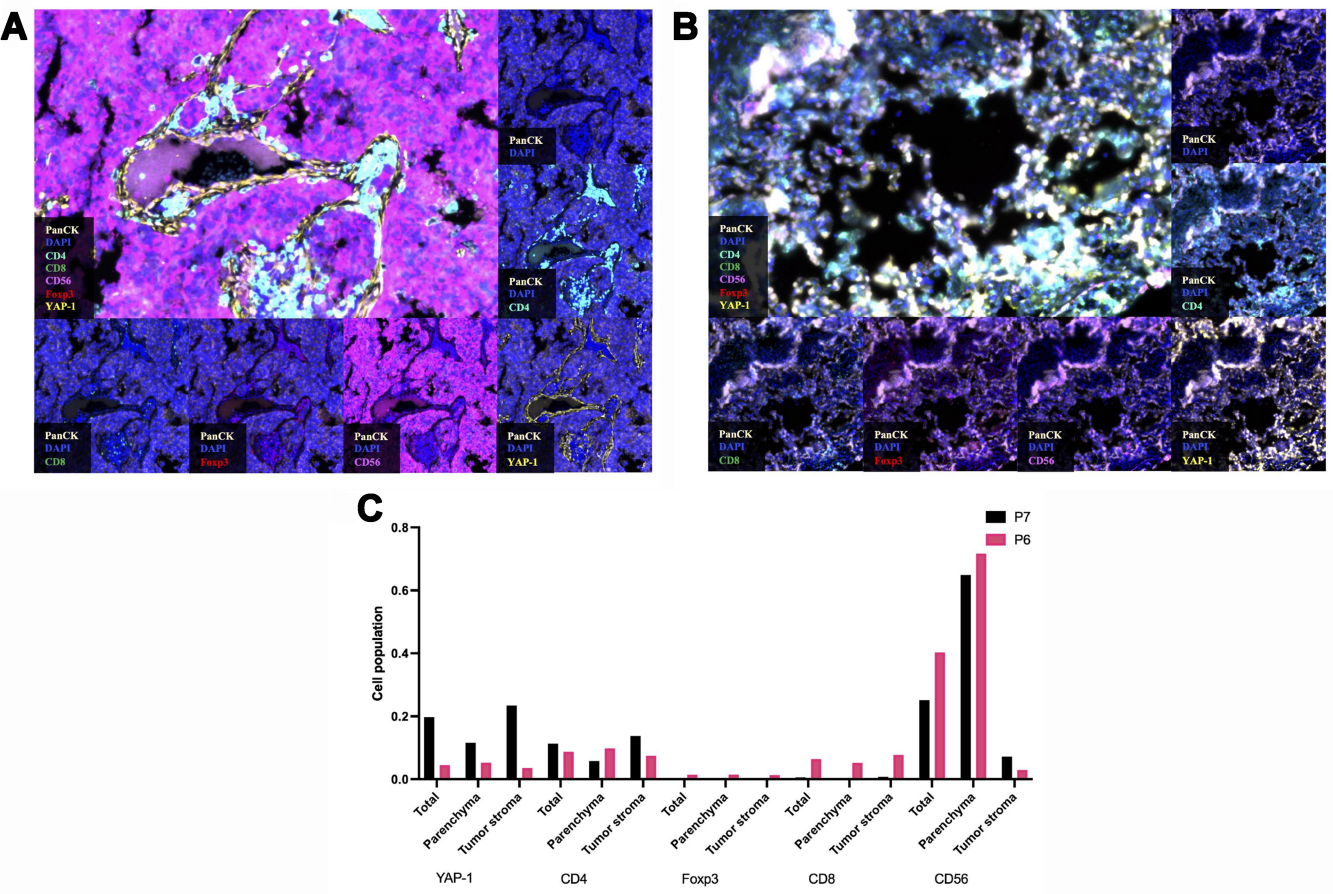
mIHC images of two representative patients showed differences in the TIME between the responder and non-responder groups. The fluorescence signal channels corresponding to each image are indicated in the bottom right corner of each figure. (A) TIME landscape of P7, who was evaluated as SD; (B) TIME landscape of P6, who achieved PR following the ECT regimen; (C) The proportion of YAP-1 positive cells and various immune cells in P7 and P6. The tumor parenchyma and stromal regions are divided based on PanCK staining. mIHC: Multi-immunohistochemistry; TIME: tumor immune microenvironment; SD: stable disease; PR: progression disease; ECT: atezolizumab plus etoposide/carboplatin.

The infiltration of immune cells in tissue slices from patients with high YAP1 expression was generally higher than that in patients with low YAP1 expression [Figure 3]. The CD56-positive cells infiltrated most in our samples, and the total population of CD56-positive cells showed a negative correlation trend with YAP1-positive cells, though this correlation was not statistically significant (*r* = -0.518, *P* = 0.05). The total population of YAP1-positive cells was significantly positively correlated with CD4-positive cells (*r* = 0.7286, *P* = 0.003). Additionally, the overall cell density of YAP1-positive cells was positively correlated with that of CD4-positive cells (*r* = 0.6931, *P* = 0.005). In the tumor parenchyma, the proportion of YAP1-positive cells was positively correlated with the proportion of CD4-positive cells and FOXP3-positive cells (*r* = 0.732, *P* = 0.004; *r* = 0.5780, *P* = 0.03). Meanwhile, the density of YAP1-positive cells in the tumor parenchyma was positively correlated with the density of CD4-positive cells and FOXP3-positive cells (*r* = 0.635, *P* = 0.02; *r* = 0.573, *P* = 0.04). However, in the tumor stroma, only CD4-positive cell density showed a significant correlation with YAP1-positive cell density (*r* = 0.690, *P* = 0.008). There was no significant correlation between the proportion or density of other immune cells and YAP1-positive cells [Figure 4].

### The prognostic value of YAP1 and TIME characteristics of SCLC was verified

**Figure 5:**
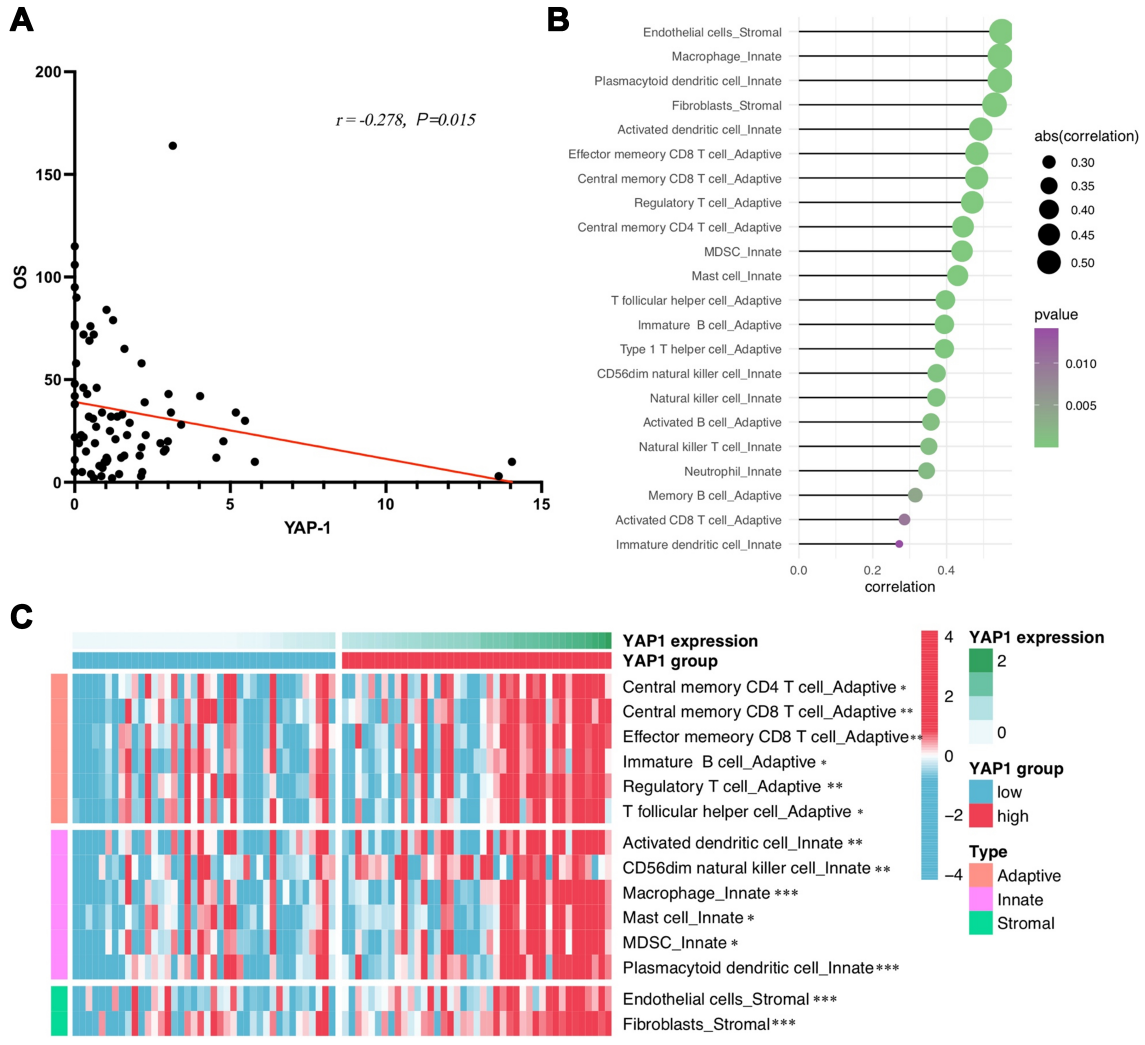
Public data analysis verified the prognostic value of YAP1 and TIME characteristics of SCLC. (A) The public data analysis showed a negative correlation between OS and YAP1 expression levels in SCLC patients; (B) The correlation between infiltration levels of different immune cells and expression level YAP1; (C) Different infiltration levels of immune infiltrating cells between YAP1-low and YAP1-high groups. (^*^P < 0.05, ^**^P < 0.01, ^***^P < 0.001). TIME: Tumor immune microenvironment; SCLC: small-cell lung cancer; OS: overall survival.

To validate our findings on the prognostic value of tumor cell YAP1 expression level and TIME for immunotherapy in ES-SCLC patients, we retrieved public data for re-analysis. As for the analysis results via public data, the expression level of YAP1 was negatively correlated with OS (*r* = -0.28, *P* = 0.015) [Figure 5A]. However, no public data on PFS of ECT were obtained; thus, no correlation analysis of PFS was conducted. We then divided all samples into two groups based on the median expression level of YAP1 in tumor cells: the YAP1-low group and the YAP1-high group. Most immune cells infiltrated at a high level in the YAP1-high group, and similar to our mIHC results, YAP1 expression levels were significantly positively correlated with central memory CD4 T cells (*P* = 0.014) and Tregs infiltration (*P* = 0.007). Additionally, the infiltration levels of T follicular helper cells were also positively correlated with the expression levels of YAP1 (*P* = 0.037) [Figure 5B and C].

## DISCUSSION

SCLC is a type of highly invasive malignant tumor, with more than half of patients being diagnosed at the extensive stage, which is associated with a poor prognosis. However, with the release of clinical research findings from IMpower133, the treatment of advanced SCLC has entered the era of immunotherapy, significantly extending patient survival. Despite this progress, the survival benefits of immunotherapy remain limited, especially when compared to solid tumors such as lung adenocarcinoma, where further improvements are still needed. In our study, we investigated the potential of YAP1 as a biomarker for predicting the efficacy of chemo-immunotherapy in ES-SCLC patients. Additionally, we explored the ES-SCLC TIME and its correlation with YAP1 expression.

In our study, the mPFS for ES-SCLC patients receiving the ECT regimen was 5.0 months, compared to 5.2 months reported for the ECT group in a clinical trial^[33]^. Meanwhile, in the IMpower133 subgroup of Japanese patients, the mPFS for the ECT group was only 4.5 months, differing somewhat from the overall study results^[34]^. A real-world study conducted in Canada reported a longer mPFS of 6.0 months for the ECT group^[35]^, whereas real-world data from South Korea showed an mPFS of only 4.6 months for ES-SCLC patients receiving ECT treatment^[36]^. These findings suggest racial disparities in the effectiveness of ECT treatment, with Asian populations appearing to derive less benefit in both clinical trials and real-world studies.

YAP1 has been reported as an indicator of poor prognosis in several cancers, including pancreatic cancer, colorectal cancer, and liver cancer^[37-40]^. In our study, a higher infiltration level of YAP1-positive cells was also related to poorer prognosis in ES-SCLC. A previous study analyzing both local and public data suggested that, compared to other subtypes, the SCLC-YAP1 (SCLC-Y) subtype exhibits the worst response to immunotherapy^[41]^. Similarly, our results indicate a negative correlation between YAP1 expression and PFS in SCLC patients undergoing immunotherapy. Moreover, we observed low YAP1 expression levels, which complicated the classification of SCLC-Y subgroups. Additionally, our results showed that YAP1-positive cells were mainly distributed in the tumor stroma of SCLC, consistent with previous research findings^[42,43]^.

In our mIHC panel, CD4 is used to locate CD4-positive helper T cells, CD8 for CD8-positive cytotoxic T cells, CD56 for natural killer (NK) cells, and FOXP3 for regulatory T cells (Tregs). Our analysis revealed that NK cell infiltration is a predominant feature of the TIME of SCLC. Although patients with high YAP1 expression exhibit greater immune cell infiltration than those with low YAP1 expression, the overall proportion of NK cells is negatively correlated with YAP1 expression. NK cells, which serve as an alternative source of cytotoxic activities, play an important role in anti-tumor immunity by counteracting immune evasion by T cells and targeting tumor cells with aberrant expression of the major histocompatibility complex (MHC) class I^[44,45]^. Additionally, NK cell infiltration has been associated with a favorable prognosis in various solid tumors^[46,47]^. As in a study involving 50 patients with lung squamous cell carcinoma, when the number of tumor-infiltrating NK cells subset marked by CD57 was higher than that of five NK cells per field, the prognosis of patients was significantly better than that of the low-level infiltration group^[48]^. Moreover, the activation of NK cells can improve the prognosis of lung cancer patients, as previously reported^[49]^. Increased infiltration of NK cells in tissues of patients with low YAP1 expression may be related to their improved prognosis. In terms of the impact of YAP1 on the TIME of SCLC reported previously, a study using 51 SCLC cell lines showed that the expression level of YAP1 is positively correlated with the degree of T cell infiltration^[50]^. These are consistent with our mIHC and RNA-seq analysis results, but there is still a lack of analysis of T cell subsets. Our results revealed positive correlations between YAP1 expression and both CD4-positive T cells and Tregs infiltration. Additionally, there was a trend of positive correlation between YAP1 expression and CD8-positive T cells. At present, some pre-clinical and clinical studies have confirmed that CD4-positive T cells have cytotoxicity that can directly kill cancer cells, but tumor killing is still attributed to CD8+T cell function^[51]^. While CD4-positive T cells subsets including TH1, TH2, TH17, TH9, follicular helper T cells and Tregs^[52]^. As reported, the high levels of infiltrating Tregs and MDSCs within tumors can inhibit the activation, expansion, and function of effector T cells, which is negatively correlated with the clinical outcomes of SCLC patients^[53]^. Moreover, in HLA class II positive tumors, the direct involvement of immunosuppressive Tregs may lead to immune evasion^[54]^. As for CD8 positive T cells, a study involving 24 ES-SCLC patients receiving first-line chemo-immunotherapy showed that the density of CD8 positive cells in the tumor stroma was significantly decreased in long-term survivors^[55]^. However, according to Qu *et al.*, there were no significant differences between the SCLC-Y subtype and other subtypes in CD8+ T cell infiltration^[56]^. Although their study did not further investigate the infiltration of T cell subpopulations or other immune cells, based on the negative prognostic role of YAP1 in ES-SCLC patients receiving ECT treatment, we infer that Tregs are the main component of CD4-positive cells in the tissues of patients with high YAP1 expression, and the poor efficacy of chemo-immunotherapy may be related to increased infiltration of Tregs.

There are some limitations to this study. As a single-center retrospective study, we are limited by sample size, and it is difficult to obtain patients’ tissue specimens due to the difficulty of surgery and biopsy. Further validation of YAP1’s chemotactic effect on CD4-positive T cells, especially Tregs, and exploration of its mechanism are ongoing. Studies with larger sample sizes to explore the TIME of SCLC, especially among the various subsets of immune cells, are warranted.

In conclusion, YAP1 has shown prognostic value of poor survival in ES-SCLC patients treated with ECT regimens. The expression of YAP1 was notably elevated and exhibited a positive correlation with Treg subsets in CD4-positive T cells, while the opposite was true in NK cells. Infiltration of YAP1-positive Tregs was associated with lower OS and PFS outcomes in ES-SCLC patients receiving immunochemotherapy. Targeting YAP1 may be a potential way to change the TIME and improve the efficacy of SCLC immunotherapy.

## DECLARATIONS

### Availability of data and materials

All data generated or analyzed in this study are included in this manuscript.

### Copyright

© The Author(s) 2025.
