# Supplementary material for: Exploring YAP1-related TIME in SCLC: implications for survival and treatment response to immuno-chemotherapy
Source: Cancer Drug Resist. 2025 Feb 17;8:8. doi: 10.20517/cdr.2024.177 (PMC11883233; doi:10.20517/cdr.2024.177)
Supplement: Supplementary file 1 [file cdr-8-8-SupplementaryMaterials.pdf]

Supplementary Materials

Exploring YAP1-related TIME in SCLC: implications for survival and treatment response to immuno-chemotherapy

Yu-Qing Chen[1,2,3,#], Jia-Xiong Tan[1,2,3,#], Ling-Ling Gao[4,#], Jia-Xing Yang[1,2,3], Jie Huang[5], Jin-Ji Yang[5], Qiang Zhao[1,2,3,6]

[1]Tianjin Medical University Cancer Institute and Hospital, National Clinical Research Center for Cancer, Tianjin 300060, China.

[2]Tianjin's Clinical Research Center for Cancer, Tianjin 300060, China.

[3]Key Laboratory of Cancer Prevention and Therapy, Tianjin 300060, China.

[4]Beihang University, Beijing 100191, China.

[5]Guangdong Lung Cancer Institute, Guangdong Provincial Key Laboratory of Translational Medicine in Lung Cancer, Guangdong Provincial People's Hospital (Guangdong Academy of Medical Sciences), Southern Medical University, Guangzhou 510080, Guangdong, China.

[6]Department of Pediatric Oncology, Tianjin Medical University Cancer Institute and Hospital, National Clinical Research Center for Cancer, Tianjin 300060, China.

[#]Authors contributed equally.

Correspondence to: Dr. Qiang Zhao, Department of Pediatric Oncology, Tianjin Medical University Cancer Institute and Hospital, National Clinical Research Center for Cancer, Ti Yuan north, Huanhu west road, Tianjin 300060, China. E-mail: zhaoqiang@tjmuch.com; Dr. Jin-Ji Yang, Guangdong Lung Cancer Institute, Guangdong Provincial Key Laboratory of Translational Medicine in Lung Cancer, Guangdong Provincial People's Hospital (Guangdong Academy of Medical Sciences), Southern Medical University, 106 Zhongshan 2nd Road, Guangzhou 510080, Guangdong, China. E-mail: yangjinji@gdph.org.cn

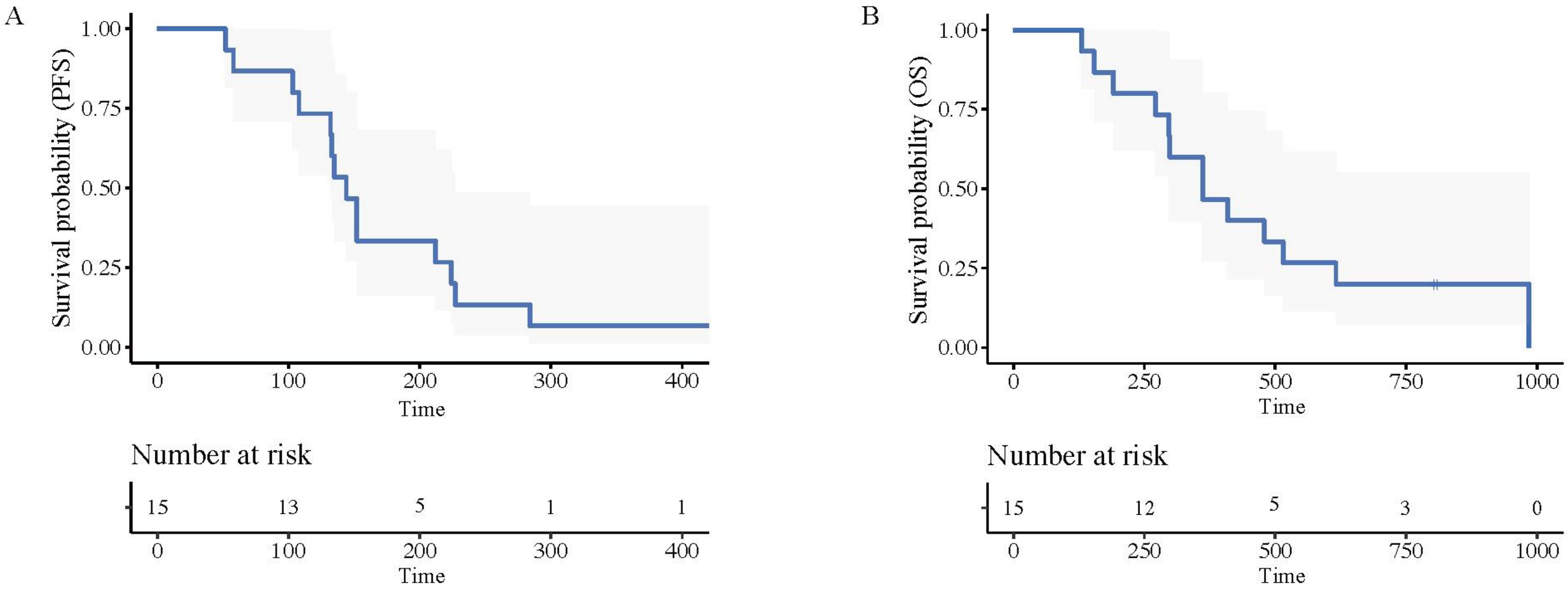

Supplementary Figure 1. K=M curves for survival analysis.

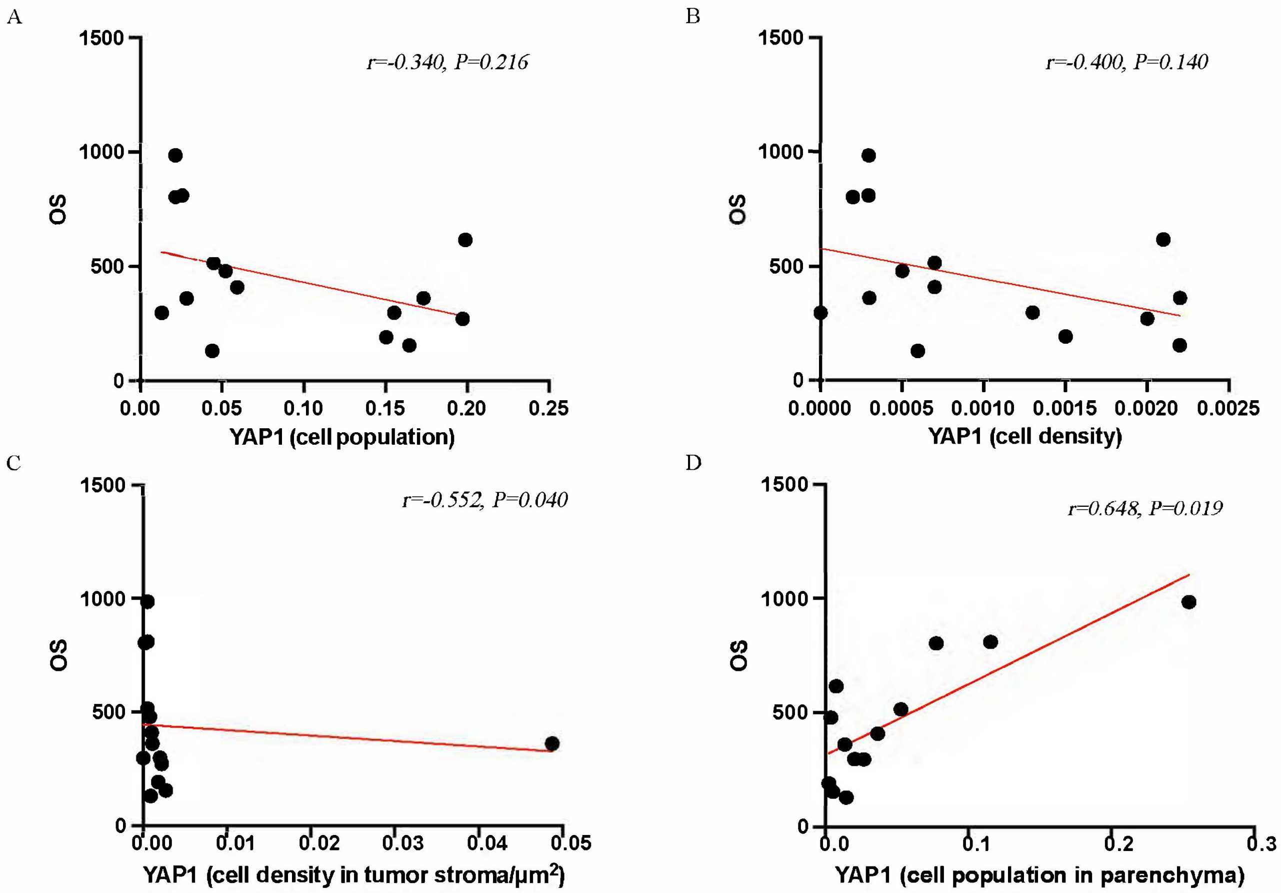

Supplementary Figure 2. Potential prognostic value of YAP1 in ES-SCLC. The linear regression fiting curve calculated by speamman correlation betwen 0S and cell population, cell density, cell density in tumor stroma and cell population in tumor parenchyma of YAP1 positive cells respectively. ES-SCLC: Extensive-stage small cell lung cancer; OS: overall survival.

Supplementary Table 1. Clinical and pathological characteristics of ES-SCLC patients received ECT regimen in this study

Patient Number	Gender	Age	Smoke (pack*years)	Confirmed Diagnosis Time	Pathology	Mix Type	NSE (ng/ml)	Ki67 (%)	Line of ECT	BOR	PFS (d)	OS (d)
1	F	66	0	2021/6/3	SCLC	NA	112.6	90	1st	PR	152	361
2	M	55	30	2020/5/26	SCLC	NA	18.47	90	2nd	SD	135	271
3	M	72	0	2020/3/18	SCLC	NA	115.1	90	1st	PR	108	515
4	M	62	100	2020/8/14	SCLC	NA	41.65	80	1st	SD	132	154
5	M	68	80	2019/3/22	SCLC	NA	40.93	90	1st	SD	284	616
6	M	66	40	2019/3/18	SCLC	NA	155	60	1st	PR	212	298
7	M	62	20	2021/2/5	SCLC Mix	LUSC	>370	90	1st	SD	103	130
8	M	70	0	2021/7/8	SCLC	NA	90.09	80	1st	SD	144	191
9	M	54	45	2022/4/12	SCLC	NA	128.6	80	1st	PD	58	297
10	M	72	80	2022/1/12	SCLC	NA	63.7	80	1st	PR	227	810+
11	M	61	17.5	2021/12/30	SCLC	NA	62.54	90	1st	PR	224	804+
12	M	59	30	2022/1/4	SCLC	NA	25.86	95	1st	PR	152	407
13	M	75	50	2022/1/7	SCLC	NA	16.13	80	1st	PR	52	479
14	M	64	80	2021/2/2	SCLC	NA	21.4	90	1st	SD	453	985
15	F	71	0	2021/12/31	SCLC	NA	49.66	80	1st	SD	133	409

ES-SCLC, extensive-stage small cell lung cancer; ECT, atezolizumab plus etoposide/ carboplatin.

Supplementary Table 2. Differences of different types of cells between the responder group and non-responder group

			Responder group		non-Responder group		*P value*
			mean	95% CI	mean	96% CI	
CD4 positive cells	cell population	total	0.1140	0.0359-0.2223	0.1152	0.0456-0.1907	0.985
		parenchyma	0.0356	0.0073-0.0672	0.0503	0.0180-0.0989	0.623
		stroma	0.1075	0.0584-0.1617	0.1662	0.0623-0.2712	0.366
	cell density	total	0.0008	0.0003-0.0013	0.0012	0.0004-0.0022	0.485
		parenchyma	0.0006	0.0001-0.0011	0.0007	0.0002-0.0015	0.704
		stroma	0.0010	0.0004-0.0017	0.0016	0.0008-0.0026	0.351
Foxp3 positive cells	cell population	total	0.0119	0.0062-0.0183	0.0162	0.0033-0.0329	0.632
		parenchyma	0.0046	0.0012-0.0092	0.0055	0.0006-0.0134	0.860
		stroma	0.0249	0.0124-0.0402	0.0240	0.0124-0.0402	0.951
	cell density	total	0.0001	0.0001-0.0002	0.0002	0-0.0003	0.778
		parenchyma	0.0001	0-0.0001	0.0001	0-0.0003	0.850
		stroma	0.0002	0.0001-0.0004	0.0002	0.0001-0.0005	0.861
CD8 positive cells	cell population	total	0.0485	0.0235-0.0813	0.0701	0.0141-0.1448	0.617
		parenchyma	0.0196	0.0117-0.0332	0.0308	0.0031-0.0784	0.672
		stroma	0.0728	0.0403-0.1082	0.0989	0.0237-0.1964	0.597
	cell density	total	0.0005	0.0003-0.0007	0.0007	0.0001-0.0017	0.608
		parenchyma	0.0003	0.0001-0.0002	0.0005	0-0.0013	0.664
		stroma	0.0006	0.0004-0.0009	0.0009	0.0002-0.0019	0.618
CD56 positive cells	cell population	total	0.3725	0.1206-0.6407	0.4700	0.3779-0.5482	0.524
		parenchyma	0.6412	0.3818-0.8778	0.7799	0.6899-0.8620	0.373
		stroma	0.1442	0.0401-0.2987	0.2216	0.1275-0.3269	0.382

	cell density	total	0.0057	0.0029-0.0085	0.0042	0.0032-0.0052	0.308
		parenchyma	0.0101	0.0053-0.0143	0.0105	0.0092-0.0120	0.853
		stroma	0.0011	0.0004-0.0022	0.0045	0.0010-0.0116	0.390
YAP1 positive cells	cell population	total	0.0351	0.0246-0.0446	0.1178	0.0680-0.1640	0.014
		parenchyma	0.0189	0.0083-0.0331	0.0737	0.0179-0.1485	0.159
		stroma	0.0182	0.0004-0.0431	0.1296	0.0348-0.2296	0.064
	cell density	total	0.0004	0.0003-0.0006	0.0013	0.0007-0.0019	0.028
		parenchyma	0.0003	0.0001-0.0005	0.0010	0.0004-0.0016	0.338
		stroma	0.0006	0.0004-0.0008	0.0075	0.0005-0.0250	0.112
